# Binder-Free Porous 3D-ZnO Hexagonal-Cubes for Electrochemical Energy Storage Applications

**DOI:** 10.3390/ma15062250

**Published:** 2022-03-18

**Authors:** Qasim Abbas, Lianghua Wen, Muhammad Sufyan Javed, Awais Ahmad, Muhammad Shahzad Nazir, Mohammed A. Assiri, Muhammad Imran, Patrizia Bocchetta

**Affiliations:** 1 Department of Intelligent Manufacturing, Yibin University, Yibin 644000, China; qasimg4u@yahoo.com (Q.A.); wlh45@126.com (L.W.); 2 School of Physical Science and Technology, Lanzhou University, Lanzhou 730000, China; 3 Departamento de Quimica Organica, Universidad de Cordoba, Edificio Marie Curie (C-3), Ctra Nnal IV-A, Km 396, E14014 Cordoba, Spain; awaisahmed@gcuf.edu.pk; 4 Faculty of Automation Engineering, Huaiyin Institute of Technology, Huai’an 223003, China; nazir@hyit.edu.cn; 5 Research Center for Advanced Materials Science (RCAMS), Department of Chemistry, Faculty of Science, King Khalid University, Abha 61413, Saudi Arabia; maassiri@kku.edu.sa (M.A.A.); miahmad@kku.edu.sa (M.I.); 6 Dipartimento di Ingegneria dell’Innovazione, Università del Salento, Via Monteroni, 73100 Lecce, Italy

**Keywords:** ZnO, porous material, binder-free electrode, hexagonal cubes, supercapacitors

## Abstract

Considerable efforts are underway to rationally design and synthesize novel electrode materials for high-performance supercapacitors (SCs). However, the creation of suitable materials with high capacitance remains a big challenge for energy storage devices. Herein, unique three-dimensional (3D) ZnO hexagonal cubes on carbon cloth (ZnO@CC) were synthesized by invoking a facile and economical hydrothermal method. The mesoporous ZnO@CC electrode, by virtue of its high surface area, offers rich electroactive sites for the fast diffusion of electrolyte ions, resulting in the enhancement of the SC’s performance. The ZnO@CC electrode demonstrated a high specific capacitance of 352.5 and 250 F g^−1^ at 2 and 20 A g^−1^, respectively. The ZnO@CC electrode revealed a decent stability of 84% over 5000 cycles at 20 A g^−1^ and an outstanding rate-capability of 71% at a 10-fold high current density with respect to 2 A g^−1^. Thus, the ZnO@CC electrode demonstrated improved electrochemical performance, signifying that ZnO as is promising candidate for SCs applications.

## 1. Introduction

In response to the growing demand for reliable and high-performance energy storage devices (ESD), the supercapacitors (SCs), also recognized as electrochemical capacitors, have garnered a lot of attention due to their attractive properties of having a high power density as well as longer cyclic life along with better safety [1]. However, SCs have a more inferior energy density than batteries. The energy density of SCs should be increased to that of a battery, which would allow them to be used as significant sources of power storage [2]. Electrochemical double-layer capacitors (EDLC) and pseudocapacitors (PCs) are two processes through which SCs store electrical energy [3]. The charge that is accumulated by means of electrode–electrolyte contact is the energy storage mechanism in EDLCs. Since carbon-based materials such as metal–organic framework (MOF)-derived carbon [4] and its derivatives have a large surface area (SA) and excellent electrical conductivity, they are commonly employed in EDLC electrodes [5]. Faradaic reactions enable PCs to deliver better capacitance and energy density than EDLC-based SCs [6]. PCs can be made with transition metal oxides and conductive polymers because they have plenty of redox sites for Faradaic reactions. Pseudocapacitive electrode materials combined with carbon materials have been helpful in increasing the capacitance of SCs [7].

Recently, pseudocapacitance has been extensively studied using transition metal oxides/hydroxides and conductive polymers. It has been revealed that ruthenium oxide (RuO_2_) has a high specific capacitance (C_sp_) in addition to showing a decent rate-performance [8]. RuO_2_, on the other hand, is quite rare and expensive. For PCs electrodes, RuO_2_ is often replaced with a variety of different transition metal oxides, including manganese oxide (MnO_2_) [9], indium oxide (In_2_O_3_) [10], cobalt oxide (Co_3_O_4_) [11], iron oxide (Fe_3_O_4_) [12], and vanadium oxide (V_2_O_5_) [13].

In electronics and piezotronics as well as in photo-catalysts and sensors, zinc oxide (ZnO) nanostructures have been explored extensively due to their distinctive physicochemical properties. Thus, a large bandgap of 3.37 eV and several technical advantages, including minimal cost, easy production, eco-friendliness, and hygienic suitability [14,15], makes it attractive to be employed as an electrode in SC. Composites such as ZnO-ZnS [16], ZnO-MnO_2_ [17], and 3DZnO-NiO [18] have been found to offer enhanced electrical conduction and good mechanical support when serving as SC electrodes [14,15]. In order to further enhance the electrical conductivity and stability of SCs, literature study reveals the preparation of ZnO composites with carbon materials. For an SC electrode, Li et al. used an electroless technique to create a nanostructured composite composed of ZnO–activated carbon (AC), which exhibited a C_sp_ of 156 F g^−1^ at 5 mV s^−1^ [19]. To make hybrid ZnO nanocomposites with graphene that can act as SC electrodes, Soheila et al. grew ZnO nanorods directly on a graphene surface that were able to deliver a C_sp_ of 187 F g^−1^ at a scan rate 5 mV s^−1^ [20]. Despite the progress made by different researchers to develop SC electrodes based on ZnO and their composites, the C_sp_ of ZnO is reported to be quite low compared with other metal oxides. Therefore, we aimed to enhance the overall super-capacitive performance of ZnO by making a binder-free electrode based on 3D-ZnO porous hexagonal cubes for the first time.

Herein, we successfully designed porous a 3D-ZnO hexagonal cube with an interconnected architecture that was homogeneously fabricated on carbon cloth (CC) using a facile and low-cost hydrothermal process. This ZnO@CC electrode possesses a large SA with mesoporous features, resulting in the provision of more electro-active sites that improve the electrochemical energy storage properties of the electrode. The ZnO@CC electrode revealed the best electrochemical performance by providing a C_sp_ of 352.5 and 250 F g^−1^ at 1 and 20 A g^−1^, respectively. The ZnO@CC electrode exhibited a reasonable stability of 84% over 5000 cycles at 20 A g^−1^. The acquired findings show the possibility of improving the electrochemical performance of ZnO-based electrodes, demonstrating its excellent prospective to be used in high-performance SC applications.

## 2. Materials and Methods

**Figure 1:**
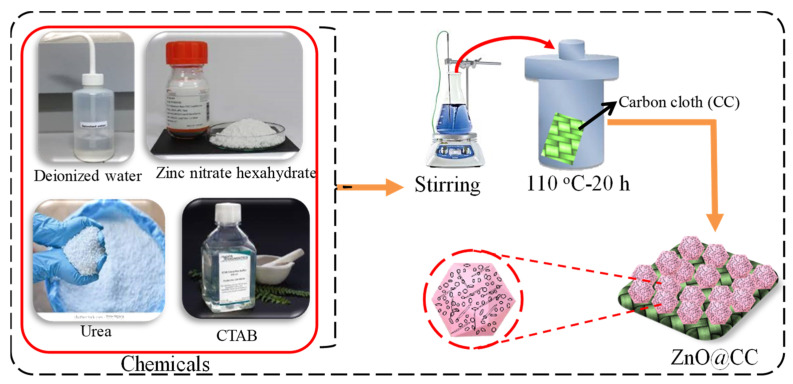
Schematic illustration for the synthesis process to create the ZnO@CC hexagonal cubes.

The simple hydrothermal approach was used for the fabrication of the ZnO@CC electrode. To obtain a homogeneous solution, Zn(NO_3_)_2_∙6H_2_O (zinc nitrate hexahydrate), CO(NH_2_)_2_(urea), and C_19_H_42_BrN (CTAB) were mixed in an mmol concentration with a ratio of 5:3:3 ratio in 60 mL deionized (DI) water and then transferred to a Teflon-lined autoclave. A pre-cleaned piece of CC was inserted in the autoclave and heated at 120 °C for 12 h. After the completion of the hydrothermal reaction, the autoclave was taken out and allowed to cool down to room temperature naturally. The ZnO covering the CC substrate was then washed using DI water and ethanol for three times to remove any residual particles. This was followed by heating the as-obtained ZnO@CC samples in an electric oven at 90 °C for 6 h, and these samples were then used for further characterizations. A schematic illustration of the synthesis process undertaken to create the ZnO@CC electrodes is demonstrated in Figure 1.

### 2.1. Characterization Techniques

The structure of the ZnO@CC electrode was evaluated by XRD (D/Max-2400, with Cu Kα radiation, λ = 1.54 Å). An X-ray photoelectron spectrometer (XPS-PerkinElmer 5000C, PHI, Kumamoto, Japan) was used to study the elemental composition of the samples and its oxidation states. The morphological studies of the ZnO@CC electrode were perceived by SEM (S-4800, JEOL, Tokyo, Japan) and TEM (JEOL, JEM-2010, Tokyo, Japan). The SA and pore size distribution study were conducted via the N_2_ adsorption/desorption experiment (BET-Tristar-3000 instrument, Micromeritics Instrument Corporation, Shanghai, China).

### 2.2. Electrochemical Measurements

The electrochemical study of the ZnO@CC electrode was performed using the CHI 760 electrochemical workstation. In order to prepare the working electrode, the sample was cut into the dimensions of 1.0 × 1.0 cm^2^, and Ag/AgCl (3 M KCl) was engaged as a reference electrode, while a Pt electrode was used as the auxiliary electrode. The aqueous electrolyte solution comprised 6 M KOH solution. Electrochemical studies were carried out by means of a cyclic voltammogram (CV), and galvanostatic charge/discharge (GCD) curves were determined at a voltage of 0.0–0.8 V. Electrochemical impedance spectroscopy (EIS) was performed in the frequency range from 0.01 Hz to 100 kHz. The C_sp_, in units of F g^−1^, was calculated using the following equation [21]:

(1) Csp=I tm V

where *I*, *t*, *V*, and *m* designate the discharge current density in the units of A, the discharge time in s, the potential window in *V*, and the mass of material in mg, respectively.

## 3. Results

**Figure 2:**
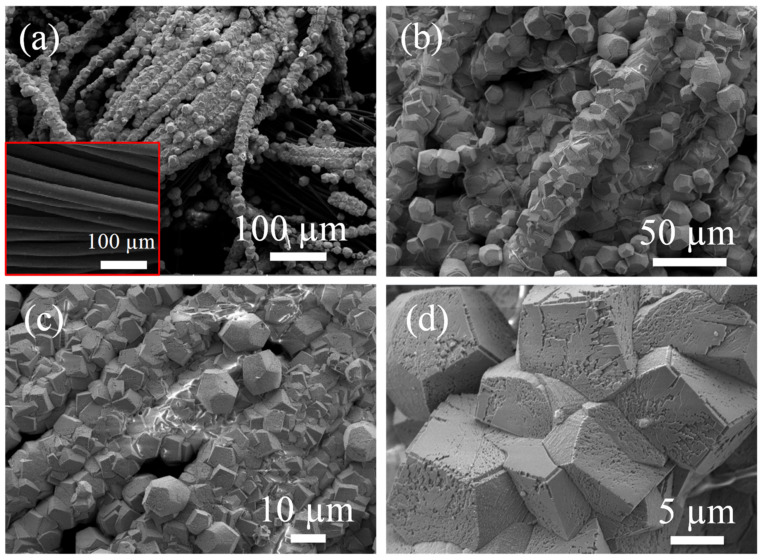
(**a**,**b**) Low- and (**c**,**d**) high-resolution SEM images of ZnO@CC.

The ZnO hexagonal cubes were primarily grown on the conductive CC substrate by engaging a facile and cost-effective hydrothermal process. Low-magnification SEM images are shown in Figure 2a,b and display the morphology of the well-aligned ZnO hexagonal cubes on the CC substrate. The inset of Figure 2a shows a SEM image of the pristine CC substrate. It can be seen that the CC substrate was entirely covered with ZnO hexagonal cubes that were approx. 8–10 μm in diameter. The dense coverage of the ZnO hexagonal cubes on the substrates is evident (Figure 2c). The high-magnification SEM micrograph (Figure 2d) shows that there are pores on the surfaces of the ZnO hexagonal cubes, which make them very advantageous for energy storage applications [22].

**Figure 3:**
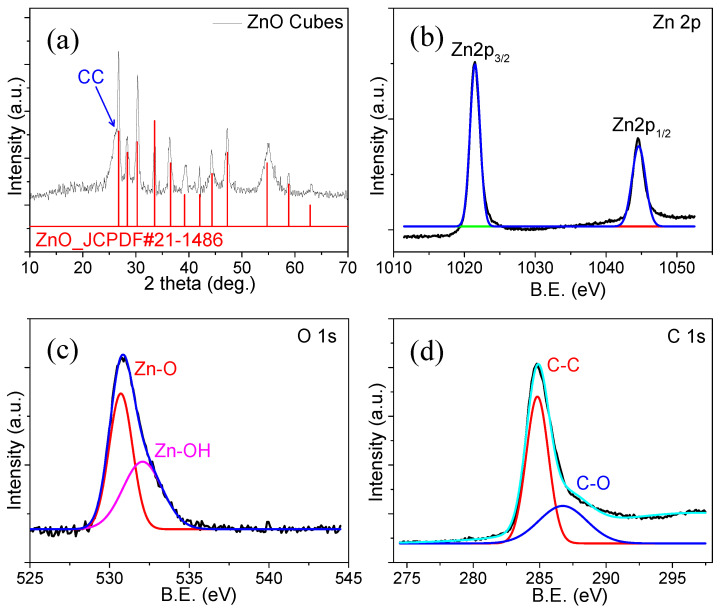
(**a**) XRD pattern, (**b**) Zn 2p, (**c**) O 1 s, and (**d**) C 1 s spectrums of ZnO@CC.

X-ray diffraction (XRD) studies on ZnO@CC were performed to probe the crystalline structure. According to the XRD pattern, ZnO crystals have a hexagonal wurtzite structure and matches the standard JCPDF #:21-1486, as shown in Figure 3a. It appears that the well-orientated ZnO hexagonal cubes on the CC without any additional impurities are indicated by intense diffraction peaks. The surface chemistry and oxidizing states of the ZnO@CC sample were studied using XPS. The Zn 2p spectrum shows two peaks at the binding energy (B.E) values of 1021.5 and 1044.6 eV for the Zn2p_3/2_ and Zn2p_1/2_ spin–orbits, respectively [23], as shown in Figure 3b. This supports the idea that the binding energies of these peaks are related to ZnO. The O 1 s spectrum exhibits two prominent Zn-O and Zn–OH peaks at B.E of 530.7 and 532.1 eV, respectively, which is shown in Figure 3c. The C 1 s spectrum exhibits two prominent C-C and C–O peaks at B.E of 284.8 and 286 eV, respectively, as demonstrated in Figure 3d.

**Figure 4:**
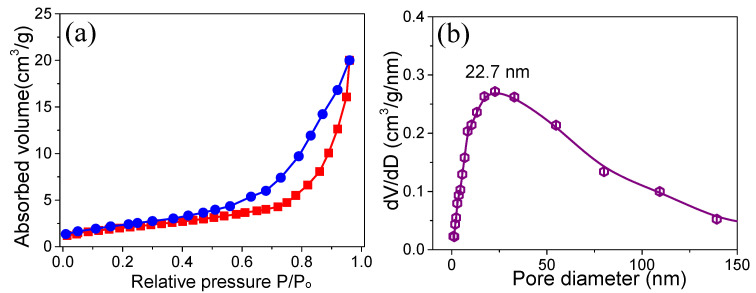
(**a**) Nitrogen adsorption and desorption isotherms and (**b**) BJH pore size distribution curves.

The surface characteristics of the ZnO were investigated using Brunauer–Emmett–Teller (BET) analysis. Figure 4a depicts the N_2_ adsorption–desorption isotherms and demonstrates a hysteresis loop at relatively higher pressure values (P/P_o_) in the range from 0.0 to 1.0. The slope increases from 0.55 to 0.96, indicating the mesoporous features. According to the plot, the highest computed SA from the BET analysis for ZnO is about 32.5 m^2^ g^−1^. The Barrett–Joyner–Halenda (BJH) pore-size distribution plot for ZnO is presented in Figure 4b. In the porous zone, the average pore radius of the material falls in the range of 22–23 nm. Porous hexagonal-shaped cubes are advantageous in SC applications because they add porosity, additional active sites, and quick electrolyte ion diffusion [24].

**Figure 5:**
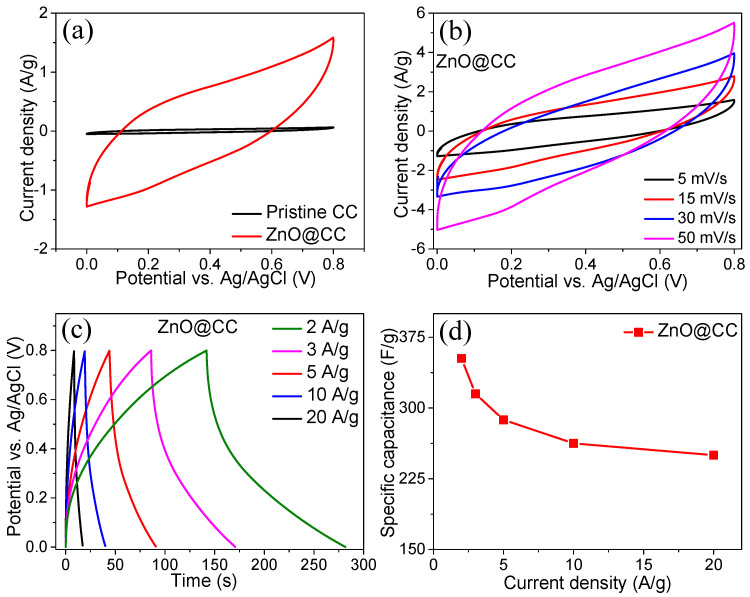
(**a**) CV curves of pristine CC and ZnO@CC, (**b**) CV curves of ZnO@CC, (**c**) GCD curves of ZnO@CC, and (**d**) Specific capacitance vs. current density.

The as-synthesized binder-free ZnO@CC electrode was directly assessed as an SC electrode. The CV curves of the ZnO@CC and pristine CC electrodes recorded at a fixed scan rate of 5 mV s^−1^ within a potential range of 0.0–0.80 V, are shown in Figure 5a. 

The area under CV curve of pristine CC is much lower than that of the ZnO@CC electrode at the same scan rate, which signifies that the charge storage contribution from the CC substrate is minimal and can be neglected. Additional CV curves for the ZnO@CC electrode were measured at various scan rates (5–50 mV s^−1^) in the same potential window, as shown in Figure 5b. Triangular-shaped CV curves without any redox peaks are observed at all scan rates, which indicates that the electrochemical performance of electrodes is originated from the capacitive process [25]. As the scan rates increased, the corresponding current densities increased, which could be attributed to the low intrinsic resistance of the electrode. The representative GCD profiles of the ZnO@CC electrode at various current densities ranging from 0.0 to 0.8 V are shown in Figure 5c. The GCD profiles confirm the typical ECDL charge storage behavior without any redox peaks and coincide with CV analysis. The GCD curves are highly symmetric and possess excellent Coulombic efficiency (~100%). The C_sp_ for the ZnO@CC electrode was determined based on the discharge time, which is displayed in discharge time versus current density graph as Figure 5d. The C_sp_ of the ZnO@CC electrode at various current densities is as follows: 352.5, 315, 287.5, 262.6, and 250 F g^−1^ at current densities of 2, 3, 5, 10, and 20 A g^−1^, respectively. It is noted that as the current density increases by 10-fold, the capacitance retention value decreases to 250 F g^−1^, demonstrating good rate capability. It can be observed from the GCD profiles that when the current density increases, the amount of decay observed in the C_sp_ values increases gradually. This behavior shows a less reversible feature of the ZnO@CC electrode because the electrolyte ions are unable to access the pores of the material effectively at a high current density. Accordingly, the capacitance decreases with the current density due to the high IR-drop [26]. This phenomenon is expected to be due to the distributed capacitance effect in the mesoporous electrodes [27]. It is worth noting here that the charge/discharge current in the ECDL capacitor is directly proportional to the scan rate. Due to the electrolyte resistance within the mesoporous structure, the IR-drop is expected to become significant at a higher current density. In addition to having an improved capacitance, the ZnO@CC electrode is superior to other electrodes, such as a ZnO/activated carbon composite with a C_sp_ of 187 F g^−1^ at a scan rate of 5 mV s^−1^ [20], ZnO nanowires with 150 F g^−1^ at 1 A g^−1^ [14], ZnO@PdO/Pd with 178 F g^−1^ at 2 mV s^−1^ [15], a ZnO/graphene composite with 156 F g^−1^ at 5 mV s^−1^ [19], and metal–organic framework-derived ZnO nanoparticles with 86.5 F g^−1^ at 2 mV s^−1^ [28].

**Figure 6:**
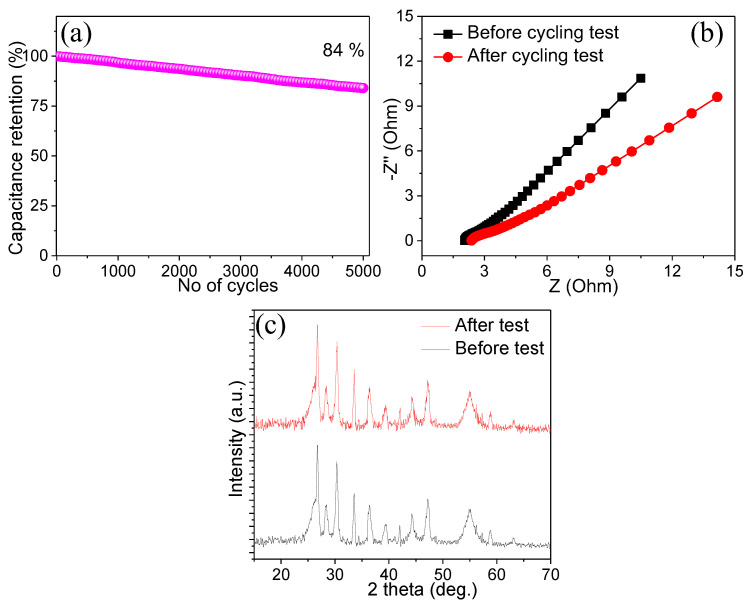
(**a**) Cycling stability test of ZnO@CC, (**b**) EIS spectra before and after cycling test, and (**c**) XRD patterns before and after stability tests.

An electrode’s long-term cycling stability is also a critical parameter for actual SC applications. Figure 6a demonstrates the repetitive charge/discharge tests at a high current density of 20 A g^−1^ with over 5000 cycles. Remarkably, the ZnO@CC electrode possesses a good cycling stability of 84%, even after 5000 cycles, which signifies that the ZnO hexagonal cubes could be a good material for energy storage applications. 

Furthermore, the EIS parameters were determined to characterize the electrochemical performance of the ZnO@CC electrode before and after the stability tests. Figure 6b illustrates the associated Nyquist plot produced in the frequency range of 0.01–100 kHz at an applied potential. In the high-frequency region, the EIS spectrum comprises a semi-arch, while at the low-frequency region, a straight line is obtained. In the high-frequency region, the intercept of the curve with the real axis refers to the resistance of the electrode material, which is denoted by R_s_, which contains the material’s inherent resistance, the ionic resistance pertaining to the electrolyte, and the interfacial resistance between the current collector and ZnO. The diameter of the semi-arch is related to the charge-transfer resistance (R_ct_) [29]. As it can be seen, the ZnO@CC electrode’s R_s_ and R_ct_ values are significantly lower, with values of 2.15 and 3.2 Ω, respectively. The R_s_ (2.32 Ω) and R_ct_ (4.9 Ω) values increased slightly after 5000 cycles, revealing the rapid conduction of electrolyte ions via the porous ZnO hexagonal cubes to the current collector during electrode testing. The EIS results indicate the improved conduction and quick diffusion of the electrolyte ions inside the ZnO@CC electrode. Furthermore, the degradation and phase changes were investigated by means of X-ray diffraction analysis before and after stability tests. This suggested that no phase changes took place during the long-term cycling test and that the material retained its original characteristics.

The outstanding electrochemical performance of the ZnO@CC electrode can be ascribed to the following reasons: (1) the mesoporous hexagonal cubes have a large SA that provides several electroactive regions for fast ion/electron transport and (2) the electrode binder-free composition and the low structural resistance of ZnO, both enable rapid electron transport during the charge/discharge process.

## 4. Conclusions

In summary, novel 3D-ZnO hexagonal cubes were synthesized directly on a carbon cloth by means of an economical and straightforward hydrothermal method. The mesoporous ZnO@CC with a high SA offers a substantial number of electroactive sites for the fast electrolytic diffusion of ions, boosting the overall performance of the SC. When used as a SC electrode, the ZnO@CC electrode presented a high C_sp_ of 352.5 and 250 F g^−1^ at 2 and 20 A g^−1^, respectively. The ZnO@CC electrode exhibited a reasonable stability of 84% over 5000 cycles at 20 A g^−1^ and superior rate-capability of 71% at a 10-fold high current density with respect to2 A g^−1^. The obtained results for the ZnO@CC electrode demonstrate improved electrochemical performance, signifying that the ZnO is a promising material for scalable and reliable SCs applications.

## Data Availability

The data presented in this study are available upon request from the corresponding author. Q.A., M.S.J. and P.B., are responsible for the experiments performed in this research.
